# Impact de la pandémie à COVID-19 sur les activités du Service de Pédiatrie du Centre Hospitalier National d´Enfants Albert Royer: étude préliminaire comparant les premiers trimestres des années 2019 et 2020

**DOI:** 10.11604/pamj.2020.36.162.23629

**Published:** 2020-07-08

**Authors:** Ousmane Ndiaye, Fatime Tall Fall, Papa Moctar Faye, Aliou Thiongane, Amadou Lamine Fall

**Affiliations:** 1 Centre Hospitalier National d´Enfants Albert Royer de Fann, Fann, Dakar,; 2 Centre d´Excellence Africain pour la Santé de la Mère et de l´Enfant de l´UCAD, Dakar

**Keywords:** COVID-19, consultations, hospitalisation, pédiatrie, Dakar, COVID-19, consultations, hospitalization, pediatrics, Dakar

## Abstract

**Introduction:**

l’objectif de cette étude est d´évaluer l´impact du COVID-19 sur les activités de soins et les recettes au Centre Hospitalier National d´Enfant Albert Royer de Dakar au Sénégal.

**Méthodes:**

il s´agissait d´une étude rétrospective, descriptive et analytique comparant les activités de consultations externes, d´hospitalisation et les recettes du premier trimestre des années 2019 et 2020.

**Résultats:**

une baisse moyenne de 33% des consultations externes a été notée au premier trimestre de l´année 2020 correspondant à la période de la pandémie comparée au premier trimestre de 2019. Une augmentation des hospitalisations était observée surtout pour les mois de janvier et février. Cependant, au mois de mars, une baisse de 11% était notée. Il en est de même pour les recettes où une baisse n´est observée qu´au mois de mars; elle était de 10%.

**Conclusion:**

l´épidémie actuelle au COVID-19 impacte fortement sur les activités de consultations externes, de soins et les recettes du centre hospitalier national d´Enfants Albert Royer. Des mesures efficaces doivent être prises pour éviter les conséquences sur la mortalité et le fonctionnement de la structure.

## Abstract (en)

**Introduction:**

the purpose of our study was to assess the impact of COVID-19 on health care activities and prescriptions at the Albert Royer National Children´s Hospital in Dakar, Senegal.

**Methods:**

we conducted a retrospective, descriptive and analytical study comparing outpatient activity, hospitalizations and prescriptions over the first-quarter of 2019 and 2020.

**Results:**

an average drop in external consultations of 33% was reported in the first quarter of 2020 (the pandemic period) compared to the first quarter of 2019. An increase in hospitalizations was observed mainly in the months of January and February. However, a drop of 11% was reported in the month of March. The same is true for prescriptions, for which a drop of 10% was reported only in the month of March.**Conclusion**: the current COVID-19 pandemic has a significant impact on outpatients´ consultations, health care activities and prescriptions at the Albert Royer National Children´s Hospital. Effective measures should be taken to prevent effects on mortality and Hospital activities.

## Introduction

L´épidémie au coronavirus SARS-CoV-2, est responsable d´une morbidité très élevée dans le monde. Elle est classée comme une urgence de santé publique par l´Organisation Mondiale de la Santé [1]. L´épicentre initialement concentré en Chine où les premiers cas ont été déclarés dans le district de Wuhan [2], s´est très rapidement déplacé aux Etats Unis et Europe, particulièrement en Italie, Espagne, en France où le plus grand nombre de cas est recensé. Les statistiques disponibles montrent une faible prévalence de la population pédiatrique et un faible taux de décès. Toutefois, très peu sont dépistés car la plupart des cas ne sont pas hospitalisés en raison d´une expression clinique moins sévère de la maladie dans cette population [3]. Selon le rapport du centre chinois de prévention et de contrôle des maladies infectieuses, parmi 72,314 cas rapportés en février 2020, seuls 2% étaient âgés de moins de 19 ans [4]. Malgré cette faible prévalence des cas chez les enfants, les services de pédiatrie sont très éprouvés en raison d´une forte stigmatisation liée à cette maladie. Une baisse importante de l´utilisation des services est observée dans la plupart des structures. Ce qui risque d´augmenter le nombre de décès extrahospitaliers liés aux autres maladies infantiles. C´est pourquoi, conformément aux recommandations de l´Association Internationale de Pédiatrie et l´Organisation Mondiale de la Santé, des mesures doivent être planifiées pour sauvegarder les activités essentielles de prévention et de soins destinés aux enfants [5-7]. Dans les pays africains, compte tenu du délai récent d´apparition de cette pandémie, aucune étude, à notre connaissance, n´est encore rapportée sur les conséquences sur les activités des services de soins infantiles. Notre étude a pour objectif d´évaluer l´impact du COVID-19 sur les activités de consultation externe et d´hospitalisation au Centre Hospitalier National d´Enfant Albert Royer de Dakar au Sénégal

## Méthodes

Ce travail a eu pour cadre le Centre Hospitalier National d´Enfants Albert Royer qui est le seul établissement public de santé (EPS) de référence de niveau 3 exclusivement pédiatrique de la région de Dakar. Il fait partie du District Sanitaire Centre. Il compte 180 lits d´hospitalisation et offre des prestations de consultation externe et d´hospitalisation. En moyenne, 5000 enfants y sont hospitalisés par an et 70000 vus en consultation externe. Il s´agissait d´une étude rétrospective, descriptive et analytique des données issues du Système d´Information Médicale (SIM). Nous avons comparé les statistiques des consultations externes du premier trimestre (janvier-février-mars) de l´année 2019 à celles de la même période pour l´année 2020 ainsi que celles des hospitalisations et des recettes financières. Nous avons calculé les variations des différentes activités et recettes entre ces deux trimestres. Les résultats ont été présentés à l´aide de diagrammes construits à l´aide de l´outil Microsoft Excel.

## Résultats

**Figure 1:**
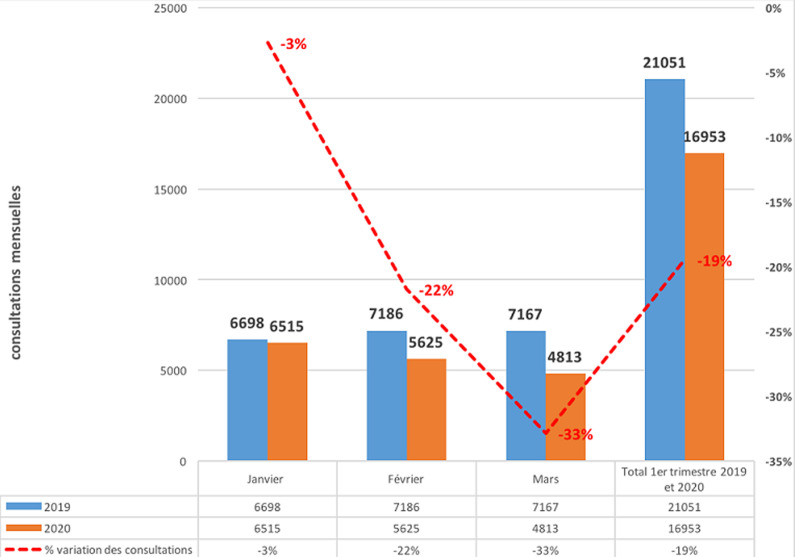
évolution des consultations externes au Centre Hospitalier National d´Enfants Albert Royer durant le premier semestre de l´année 2019 et le premier semestre de l´année 2020 au Centre Hospitalier National d´Enfants Albert Royer

**Figure 2:**
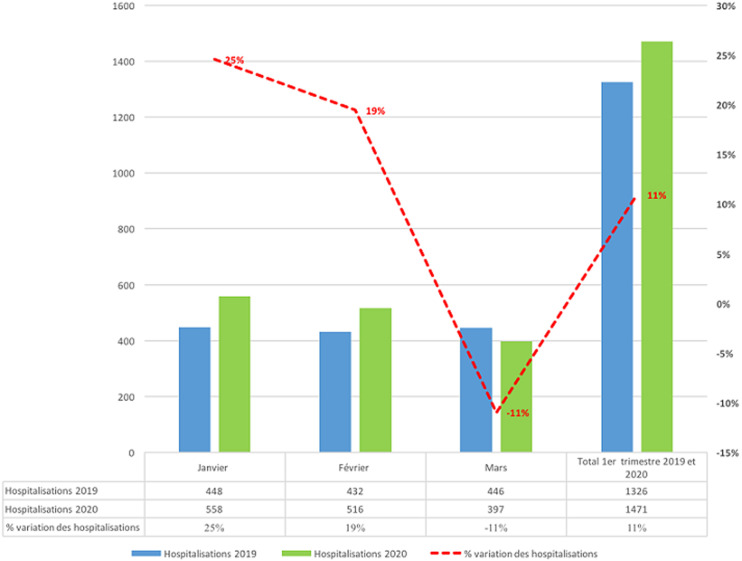
évolution des hospitalisations en pédiatrie durant le premier semestre de l´année 2019 et le premier semestre de l´année 2020 au Centre Hospitalier National d´Enfants Albert Royer

**Figure 3:**
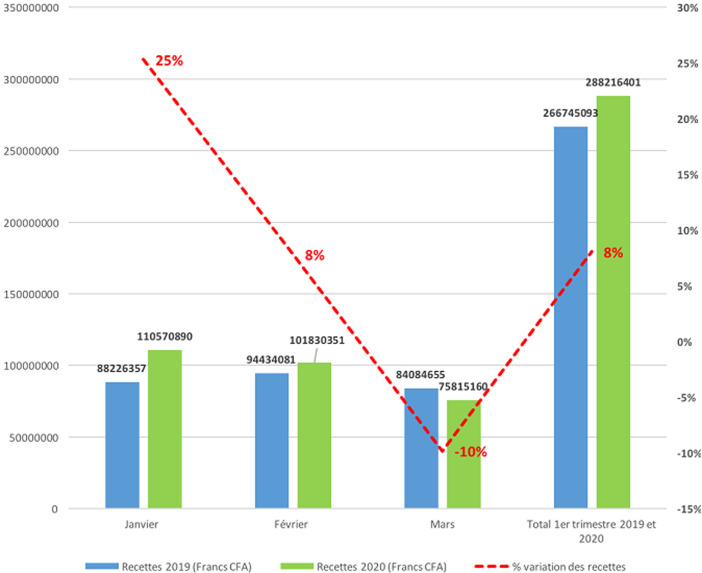
évolution des recettes au centre Hospitalier National d´Enfants Albert Royer durant le premier semestre de l´année 2019 et le premier semestre de l´année 2020 au Centre Hospitalier National d´Enfants Albert Royer

La Figure 1 montre une baisse progressive des activités de consultation externe de 3% en janvier, 22% en février et 33% au mois de mars, correspondant à une baisse moyenne de 19% pour le premier semestre 2020 comparé au premier semestre de l´année 2019. Concernant les hospitalisations, on note plutôt une augmentation durant la même période de 11% sur les effectifs agrégés. Cependant, elle n´est présente que pour les mois de janvier et février alors qu´au mois de mars 2020, nous notons une baisse de 11% des hospitalisations (Figure 2). Quant aux recettes financières, elles ont connu une augmentation malgré la baisse constatée des activités de consultation externe (-19% en moyenne) et d´hospitalisation (-11% entre février et mars); une augmentation de 8% en moyenne est notée si on compare les premiers trimestres des années 2019 et 2020 (Figure 3).

## Discussion

Les stratégies mises en œuvre au niveau de nos pays pour atteindre les objectifs du millénaire pour le développement liés à la santé avaient permis de faire des progrès remarquables dans la réduction de la mortalité des enfants de moins de 5 ans [8,9]. Cependant, de sérieuses menaces pèsent sur ces acquis compte tenu du contexte actuel lié à la pandémie au nouveau coronavirus 19. En effet cette dernière, risque d´impacter très fortement nos faibles économies mais également la continuité des activités au niveau des points de prestation de soins sur toute l´étendue de la pyramide de santé. Au niveau des structures de santé de référence, les effets risquent d´être très marqués. Ainsi, comme le démontrent les résultats de notre étude comparant les activités de consultation externe et d´hospitalisation des premiers semestres de deux années consécutives (2019 et 2020), nous notons une perturbation progressive des activités de soins au service de Pédiatrie de l´hôpital d´Enfants Albert Royer de Dakar. Le premier trimestre de l´année 2020 correspond à l´entrée et l´intensification de l´épidémie dans la plupart des pays africains au Sud du Sahara, au Sénégal en particulier. Une réduction moyenne de 19% des consultations a été constatée signifiant une baisse importante de la fréquentation des services; cette dernière était plus marquée au mois de mars 2020 avec 33% de baisse. Ceci pourrait s´expliquer par la peur de l´exposition au virus ncov19 dans les structures de soins infantiles pour les familles et le personnel soignant souvent mal protégés, aux contraintes liées à la restriction des déplacements décidée par les autorités de nos pays et une réorientation des activités cliniques au profit de la lutte contre l´épidémie actuelle au détriment des activités de soins traditionnels [10, 11]. Chang HJ *et al*. [12], dans une étude réalisée à Taiwan, rapportait une baisse de 23,9% des activités de soins ambulatoires et de 35, 2% des hospitalisations au cours de l´épidémie à SARS de 2003. Quant à Brolin Ribacke KJ *et al*. [13], ils notaient une diminution de 27,6% de l´utilisation des services et 44,3% des hospitalisations au cours de l´épidémie à Ebola en Afrique de l´Ouest.

Dans une étude récente de modélisation portant sur les effets indirects de la pandémie au COVID-19 sur la mortalité maternelle et infantile dans les pays en développement, Roberton T *et al*. [14] prédisent une hausse de la mortalité des enfants de moins de 5ans de 9,8% à 44,7% par mois qui pourrait être liée en partie à la baisse de la couverture en soins infantiles; celle-ci serait estimée entre 14,3% et 49,4%. Dans notre étude nous notons une augmentation globale des hospitalisations durant le premier trimestre de l´année 2020 comparée à l´année 2019. Ce qui semble paradoxal eu égard à la situation épidémiologique actuelle. Cependant elle pourrait s´expliquer par les raisons suivantes: l´offre de service a été améliorée au cours du deuxième semestre de l´année 2019 par la création d´un service d´accueil des urgences (SAU) et la réception d´un nouveau service de néonatologie de 60 lits correspondant à une capacité trois fois plus importante que celui ayant fait l´objet d´une rénovation. Quant aux recettes, elles apparaissent augmentées comparées à l´année 2019. Cette hausse est sans nul liée à l´augmentation des capacités litières d´hospitalisation mais également par une revue de la tarification de tous les actes médicaux décidés par le conseil d´administration de l´hôpital qui a un statut d´établissement publique de santé de niveau 3 (EPS 3) dotée d´une autonomie de gestion. Toutefois, une analyse plus fine des hospitalisations et des recettes permet de mettre en évidence une baisse respectivement de 11% et 10% au mois de mars; le premier cas de COVID-19 ayant été diagnostiqué au début du mois de mars au Sénégal. Ce qui démontre l´impact très rapide sur les activités. La situation pourrait être pire si la pandémie perdure.

## Conclusion

La pandémie actuelle au COVID-19 risque d´impacter négativement sur les prestations de service destinées aux enfants dans les pays en développement en général, au Sénégal en particulier. A l´hôpital Albert Royer, une baisse progressive des activités durant le premier trimestre suivant son début a été constaté. Pour une plus grande résilience de nos structures de soins, les mesures suivantes devraient être prises: faire un plaidoyer pour un maintien et une amélioration de la qualité des services de soins infantiles; mettre en place des moyens suffisants de prévention contre le COVID-19 au niveau des structures hospitalières afin de sécuriser les professionnels de santé et redonner confiance aux familles; renforcer les système de santé infantile dans le cadre des plans d´investissement post-COVID-19.

### Etat des connaissances sur le sujet

- La pandémie à COVID-19 risque de perturber les activités des unités de prise en charge des enfants;
- Un risque élevé d´augmentation de la mortalité intra et extrahospitalière existe et pourrait annihiler les efforts faits pour réduire la mortalité infantile.

### Contribution de notre étude à la connaissance

- Notre étude confirme l´impact du COVID-19 sur les activités des établissements public de de santé de référence en soins pédiatriques;
- On note une baisse de la fréquentation hospitalière et une baisse des recettes financières qui peut compromettre les salaires des agents de santé et retentir sur la qualité de la prise en charge;
- Nous attirons l´attention des autorités pour la prise en compte de ces contraintes dans l´appui à apporter aux établissements publics de santé impactés.

## Remerciements

Nous remercions l´administration, l´équipe du système d´information médicale et le responsable financier du Centre Hospitalier National d´Enfants Albert Royer de Dakar qui ont mis à notre disposition les informations médicales et administratives.
